# Results from PSIPROSPER: A multicenter retrospective study to analyze the impact of treatment with paliperidone palmitate 1-month on clinical outcomes and hospital resource utilization in adult patients with schizophrenia in Portugal

**DOI:** 10.3389/fpsyt.2022.992256

**Published:** 2022-11-01

**Authors:** João Marques-Teixeira, Gonçalo Amorim, Ana Catarina Pires

**Affiliations:** ^1^Neurobios–Instituto de Neurociências, Porto, Portugal; ^2^Janssen-Cilag Farmacêutica, Oeiras, Portugal

**Keywords:** schizophrenia, paliperidone palmitate 1-month formulation, oral antipsychotics, treatment, clinical outcomes, healthcare resources

## Abstract

**Background:**

Schizophrenia is a chronic psychiatric disorder with a significant impact worldwide. The early onset and its relapsing nature pose a significant challenge to patients and caregivers. The PSIPROSPER study aimed to characterize the real-world context of schizophrenia treatment in Portugal and to measure the impact of including paliperidone palmitate 1-month formulation (PP1M) in the clinical outcomes (relapses and hospitalizations) and healthcare resource utilization, in a context in which payment scheme in Portugal allows for patients to receive free antipsychotics if prescribed at public hospitals.

**Methods:**

This was a multicenter, retrospective, observational study. Male and female adults with a diagnosis of schizophrenia who initiated treatment with PP1M after a minimum of 12 months on an Oral Antipsychotic (OAP), and with complete medical charts, were consecutively included. A mirror-image design over 24 months allowed the comparison of outcomes before and after the PP1M introduction.

**Results:**

Out of the 51 patients included, 80.4% were male, with a mean age of 34 (±9.8) years. Around 92% of patients were being treated with PP1M at inclusion. Lack of adherence to previous OAP was the main driver for PP1M initiation. Only 9.8% of patients were hospitalized during the PP1M period vs. 64.7% during the OAP period (*p* < 0.0001). The mean number of hospitalizations (0.1) was significantly lower during the PP1M period (*p* < 0.0001). Type of treatment was the only variable found to be significant in predicting a lower hospitalization rate and a lower risk of hospitalization. Relapses were significantly lower (*p* < 0.0001) in PP1M (21.6%) vs. OAP (83.7%). Similarly, the mean change in the number of relapses (*p* < 0.0001) showed significantly better outcomes in PP1M.

**Conclusion:**

This study supports PP1M as part of schizophrenia treatment in Portugal. Given the lower number of relapses and hospitalizations observed in schizophrenia patients treated with PP1M when compared to OAP-treated patients, this real-world study seems to provide further evidence to support the use of PP1M to treat this condition, in line with previous research. In the context of scarce public resources, these benefits should be carefully considered by healthcare decision-makers to ensure optimal value-based treatment strategies.

## Introduction

Schizophrenia is a chronic burdensome psychiatric disorder affecting roughly 20 million people worldwide (1), with high prevalence—around 1% lifetime prevalence—and increasing incidence—to roughly 1.13 million cases in 2017 (2). Age of onset is typically in adolescence and early adulthood, with male patients showing a higher prevalence and an earlier onset of the disease (3, 4). This condition is characterized by multiple relapses and psychotic episodes which have a significant detrimental impact on the life of patients and their families—impacting the performance in work and/or academic-related tasks (5, 6) and quality of life (7, 8). Relapses are common in schizophrenia, especially during the first years of treatment (9–11). Also, schizophrenia can pose a significant strain on public healthcare services, as a result of a high rate of hospitalizations and indirect costs (12, 13).

As in other mental disorders, the outcome and impact of schizophrenia can be positively influenced by early diagnosis and the subsequent treatment initiation (1, 14–16). The treatment for schizophrenia has improved significantly in recent times. The World Federation of Societies of Biological Psychiatry recommends that treatment should be initiated with antipsychotics, and the therapeutic class should be adjusted to each patient’s profile (17). The main driver is to ensure treatment compliance, adherence, and long-term maintenance of the treatment without interruptions. Poor treatment adherence and compliance with oral antipsychotics (OAPs) reportedly lead to high relapse rates, which translates into poor clinical outcomes and a high economic burden (18). A qualitative study’s systematic review on the experience of patients with LAIs showed that patients’ education is essential, as well as a close therapeutic context with the attending physician, to increase patient adherence to such therapeutics (19). In the cases where this happens, the overall subjective experience of patients is deemed as very positive (9–11, 18).

In Portugal, there are several approved treatments for schizophrenia. Paliperidone palmitate 1-month formulation (PP1M) is reimbursed since January 2014 and is administered every month. An economic analysis conducted in Portugal revealed that PP1M is more cost-effective than OAPs and more beneficial than other long-acting injectable antipsychotics (LAIs) (haloperidol decanoate and risperidone microspheres) in terms of quality-adjusted life years (QALYs), relapse, and hospitalization rates (18). Other studies have shown that PP1M provides good outcomes in relapse prevention and hospitalizations. This is even more significant in patients with prior history of non-adherence to treatment, which is also highlighted by the reduction in bed use in fully compliant patients under PP1M (20, 21). LAIs have shown better outcomes in a real-world setting—namely in hospitalizations and relapse risk—compared to OAPs (22), with lower rates of treatment failure. In addition, landmark studies have shown that PP1M has better treatment outcomes than OAPs, with a longer time until relapse (23, 24). Overall, LAIs seem to provide better results in the risk of death compared to OAPs, which is also a key factor in mental health (25). Beyond benefits in clinical outcomes, PP1M has shown benefits in subjective wellbeing, which is certainly associated with the rapid action of PP1M, showing a significant therapeutic effect 8 days after treatment initiation (26). A study in Romania conducted during the COVID-19 pandemic showed that during this period, there was a significant decrease in the initiation of LAIs, which is expected to have a negative impact on relapses in the short term (27). Conversely, a study in Canada did not show such trends during the pandemic, in which the number of initiations, switching, and discontinuations remained stable (28). Another study from Switzerland showed that the prescription of LAIs only happened in around 14% of schizophrenia spectrum disorder patients eligible for such therapeutics (29). In Spain, the trend seems to suggest that LAIs are becoming an increasingly available therapeutical option among physicians (30). Currently, data are scarce in terms of therapeutic management of schizophrenia in the real-world setting in Portugal since the introduction of PP1M. Moreover, recent changes concerning the payment of antipsychotics are expected to have an impact on prescriptions, since patients are no longer required to pay if the treatment is prescribed by the attending physician at a public hospital. This contributes to centering the clinical decision on the specific needs of the patient, without considering the purchasing power of patients. We conducted a retrospective study to assess the impact of 1-year treatment with PP1M on relapses and hospitalizations in adult patients with schizophrenia without treatment resistance compared to a mirror period of patients receiving OAPs.

## Materials and methods

### Study design and population

PSIPROSPER was a multicenter, retrospective, observational study primarily designed to analyze the impact of PP1M treatment on clinical outcomes and use of healthcare resources—with emphasis on hospital-resources utilization. The study was conducted following each site’s routine clinical practice in the management of schizophrenia. Overall, 20 sites participate in the study (Supplementary Table 6). Sites were selected as these were public hospitals—reference regional sites for the treatment of psychiatric conditions—covering all the main regions in Portugal.

**FIGURE 1:**
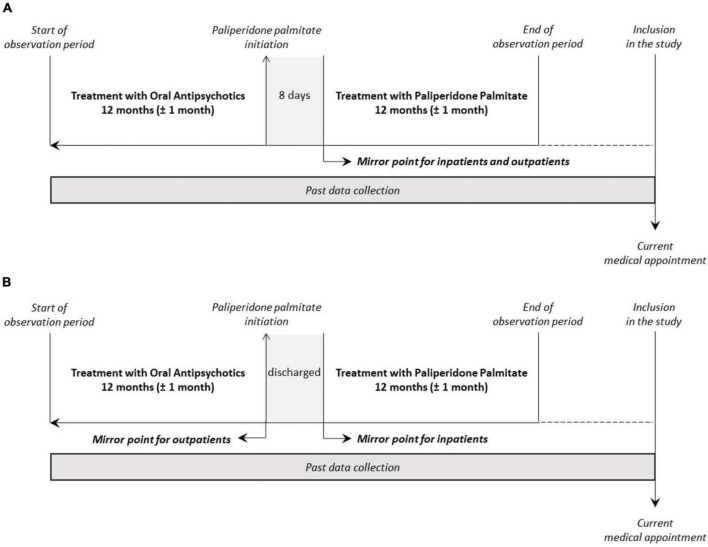
Study design: main **(A)** and sensitivity **(B)** analyses. **(A)** Main analysis: the mirror point of the study was set at 8 days after treatment initiation for patients that started PP1M in the community (outpatients) and for those that started PP1M during a hospitalization (inpatients); **(B)** sensitivity analysis: schematic representation of the sensitivity analysis that considers the mirror point for outpatients as the date of PP1M initiation, and for the inpatients as the date of the hospitalization discharge.

The study design also included an observation period of 2 years (24 months) as depicted in Figure 1. The mirror-image design is being used for this study to promote outcomes comparison before and after the initiation of treatment with PP1M. A mirror point had to be set as 8 days after PP1M initiation for patients that started PP1M in the community (outpatients) and the same for those that started PP1M during a schizophrenia-related hospitalization (inpatients). The reason for such a period is based on available literature that states that this is the treatment period required for PP1M to achieve a clinically meaningful response (24, 26). This option is to control for hospitalizations that may occur during the transition from OAP to the PP1M treatment, which can be attributed to the efficacy failure of OAP treatment (31). The start of the observation period is defined as 12 months before the designated mirror point (±1 calendar month), while the end of observation (end-of-study—EOS) was considered as 12 months after the mirror point (±1 calendar month), totaling 24 months in total.

The first-patient-in occurred in September 2018 and the last visit was in April 2021. The study population consisted, based on the inclusion criteria, of male and female adult (18 and 55 years) patients with a diagnosis of schizophrenia—founded on ICD-9 criteria, which was the current ICD version used for the duration of the study in the Portuguese National Health System (NHS), or the corresponding ICD-10 codes if applicable— who initiated PP1M therapy (either switched from OAPs to PP1M, and/or added PP1M to oral therapy), and with available information concerning the annual schizophrenia-related hospitalizations prior and after initiation of PP1M treatment in their medical charts. Patients with treatment-resistant schizophrenia—at least two failed adequate trials with different antipsychotics (at maximal antipsychotic efficacious label dose for 4 to 6 consecutive weeks)—and patients with a history of treatment with clozapine, who participated in a clinical trial during the observation period or who switched to PP1M before the 31 July 2014 or who were treated with other LAIs in the 12 previous months before the observation period, were excluded as per the protocol exclusion criteria. Patients were previously treated with OAP for at least 12 months before switching to PP1M. During the treatment with OAPs, dose adjustments were allowed. Patients should have been treated with PP1M for at least 4 consecutive months after the switch.

Eligible subjects were enrolled consecutively according to routinely scheduled medical appointments until the target sample size per site was achieved. All patients or their legal representatives provided written consent to participate in this study. As per local legislation, the study was approved by each site’s ethics committees, which implies that the study did not receive nationwide approval from a specific regulatory body. Due to some limitations in the sites, the study duration had to be extended to achieve the recruitment target.

Patients were assured that the decision to participate in this study would not involve, in any way, a change in their routine treatment and disease management defined as the standard-of-care or any benefits to which they were previously entitled. Only data available per clinical practice were collected within this study.

### Data and assessments

This study included a cross-sectional data collection—at the moment of the inclusion in the study—to gather updated information regarding the use of PP1M treatment, during the patient’s routine clinical appointments. Data regarding patient characterization, sociodemographic and disease characteristics, comorbidities, medications (OAPs and co-medications), and healthcare utilization (hospitalization, emergency admissions, and outpatient psychiatric consultations) were collected from the patient’s medical records available at the sites. Schizophrenia was classified using the ICD-9/ICD-10 codes.

Relapse was defined as the re-emergence of psychotic symptoms of schizophrenia requiring:

- Psychiatric hospitalization due to worsening symptomatology (not for social reasons) as determined by the investigator; or
- An increase in the level of psychiatric care required by the subject (e.g., significant crisis intervention needed to avert hospitalization, clinically notable increases in the frequency, or intensity of subject contact required to maintain outpatient status); or
- Deliberate self-injury, suicidal, or homicidal ideation that is clinically significant as determined by the investigator, or violent behavior resulting in clinically significant injury to another person or property damage.

### Statistical analysis

All quantitative variables were summarized using descriptive statistics namely the number of valid values, mean, median, quartiles (Q1 and Q3) standard deviation and range (minimum and maximum), and categorical variables through absolute (n) and relative frequencies (%). Additionally, 95% confidence intervals were presented whenever of interest.

All statistical analyses were performed based on the Full Analysis Set (FAS).

Given the retrospective nature of the study, no imputation of missing data was performed for the secondary analyses. All statistical tests were two-sided with statistical significance set at *p* < 0.05. Statistical analysis was conducted through the software SAS^®^ (version 9.4; SAS Institute Inc., Cary, NC, United States).

For the main analysis, related to primary objectives, the mirror point of the study was set at 8 days after treatment initiation for patients that started PP1M in the community (outpatients) and for those that started PP1M during a hospitalization (inpatients). As for the sensitivity analysis, a schematic representation of the sensitivity analysis that considers the mirror point for outpatients as the date of PP1M initiation and for inpatients as the date of the hospitalization discharge is provided. In addition, a sensitivity analysis was performed considering the mirror point for outpatients as the date of PP1M initiation, and for the patients that initiated PP1M during hospitalization as the date of that hospital discharge.

Patient’s treatment history and healthcare resources used were presented by treatment (before vs. after PP1M initiation). Changes in the mean number of hospital admissions in patient subgroups, mean length of hospitalizations, mean cumulative length of hospitalization, and mean number of psychiatric emergency admissions were analyzed through paired *t*-tests or Wilcoxon tests, whenever the normality assumptions were violated. The proportion of patients hospitalized with at least one schizophrenia-related hospitalization, the proportion of outpatient psychiatric consultations, and the proportion of relapse were compared between both treatments through McNemar’s test (MN). Moreover, 95% confidence intervals were presented for each treatment. The healthcare resources endpoints were performed for 12 months before and after the initiation of PP1M treatment.

A generalized linear model (without any predictors), with Poisson distribution for counts with a logarithm of individual time as an offset variable was used to measure the global hospitalization rate. A generalized estimating equation model, with Poisson distribution for counts assuming a correlation structure and the logarithm of individual time as an offset variable, was used to examine the relationship between various independent variables (sex, age, type of treatment, number of OAPs, duration of OAPs, and concomitant medications) and the risk of hospitalization outcome, as measured by the individual hospitalization rate. The initial model (model with all variables of interest) and the optimized model (model with statistically significant variables selected by backward elimination) were presented with the corresponding 95% CI and *p*-Values, whenever applicable. The number of patients contributing to each model was presented.

An exploratory logistic regression model based on a generalized estimating equation was used to examine the relationship between various independent variables (sex, age, type of treatment, and risk factors) and the odds of hospitalization outcome, as measured by binary variable. The initial model (model with all variables of interest) and the optimized model (model with statistically significant variables selected by backward elimination) were presented with the corresponding 95% CI and *p*-Values. The number of patients contributing to each model was presented.

## Results

### Patient characteristics

A total of 51 patients were enrolled, of whom 13 (26.0%) patients were treated with PP1M in monotherapy and 37 (74.0%) were concomitantly treated with OAP. The available data namely pertaining to treatment dates did not allow us to determine if one of the patients concomitantly received OAP and PP1M. Most patients (*n* = 29, 59.2%) initiated PP1M during hospitalization (inpatients), while 20 (40.8%) initiated PP1M in the community (outpatients). Two patients were not classified due to incomplete dates. At the end of the observational period with PP1M, 32.3% (*n* = 20) were also treated with an OAP.

The patient’s mean age was 33.7 (±9.8) years with a predominance of male patients (*n* = 41, 80.4%).

The mean disease duration at the start of PP1M was 4.9 years (*n* = 17), and the median time from first symptoms to diagnosis was 1.1 years (*n* = 8). Schizophrenia was classified using the ICD-10 codes for 56.9% of the patients and using the ICD-9 codes for 43.1%, according to routine clinical practice. Most patients had a classification of paranoid schizophrenia (ICD-9−72.7%, ICD-10−55.2%), Table 1.

**TABLE 1:** Sociodemographic characteristics and disease history at the start of the observation period.

	Total (*n* = 51)
**Age (years)**	
Mean (SD)	33.7 (9.7)
Min-Max	20−55
**Gender, n (%)**	
Male	41 (80.4%)
Female	10 (19.6%)
**Duration of disease at start of PP1M (years)**	
N	17
Mean (SD)	4.9 (4.2)
**Time between first symptoms and diagnosis (years)**	
N	8
Median	1.1
Min-Max	0.01−7.5
**ICD codes used for type of schizophrenia, n (%)**	
ICD-9 codes	22 (43.1%)
ICD-10 codes	29 (56.9%)

SD, standard deviation; Min, minimum; Max, maximum. Variables related with comorbidities and psychiatric comorbidities had a different number of missing values and percentages were calculated based on non-missing information.

Hypercholesterolemia (*n* = 14, 35%), hyperprolactinemia (*n* = 6, 21.4%), and obesity (*n* = 6, 21.4%) were the most prevalent comorbidities, while smoking habits (*n* = 20, 48.8%) and alcohol/drug abuse (*n* = 20, 48.8%) were the most frequently reported psychiatric comorbidities. Most patients with smoking habits were current smokers (*n* = 17, 85%), whereas, in patients with alcohol/drug abuse, 70.0% (*n* = 14) reported it as a past event (Supplementary Table 1).

### Characterization of treatment during the observation period

At the start of the observation period, 3 (5.9%) patients had first OAP therapy ongoing (Table 2). The OAP was mostly administered once daily (*n* = 35, 71.4%), while the most frequent reason to start OAP treatment was “First antipsychotic treatment” (*n* = 19, 37.7%), in line with the characteristics of routine medical practice in Portugal.

**TABLE 2:** Treatment characterization and OAP treatment at the inclusion visit.

	Total (*n* = 51)
**Current treatment, n (%)**	
Paliperidone palmitate	47 (92.2%)
Other	4 (7.8%)
**If none or other:**	
Previous treatment with PP1M, n (%)	
Yes	4 (100%)
**Treatment duration at inclusion visit (months)**	
N	4
Median	11.62
Min-Max	3.9−98.6
**Reason for discontinuation of PP1M treatment, n (%)**	
Lack of adherence	2 (50.0%)
Patient’s choice	1 (25.0%)
Unknown	1 (25.0%)

	**Treatment with OAP**
	**n (%)**

**First ongoing OAP therapy at start of observation (period)**	
n (%)	
Yes	3 (5.9%)
**Duration of treatment (months)**	
N	23
Median	15.87
Min-Max	0.26−82.21
**Frequency of administration, n (%)**	
Once daily	35 (71.4%)
Twice daily	12 (24.5%)
3 times daily	2 (4.1%)
**Reason to start OAP treatment, n (%)**	
First antipsychotic treatment	19 (37.3%)
Lack of efficacy and adherence of the previous treatment	5 (9.8%)
Lack of efficacy of the previous treatment	4 (7.8%)
Lack of adherence of the previous treatment	3 (5.9%)
Unknown	15 (29.4%)
Other*	5 (9.8%)

nTrts, number of treatments; OAP, oral antipsychotics.

*This includes “patient choice”, “safety or tolerability reasons of the previous treatments”, and “other reason”.

Approximately 92% of patients were treated with PP1M at inclusion. Only 7.8% (*n* = 4) of the patients were under other treatment at inclusion. These patients had previous treatment with PP1M but this was discontinued due to the following reasons: lack of adherence (*n* = 2, 50%), patient’s choice (*n* = 1, 25%), or unknown reason (*n* = 1, 25%) (Table 2).

Lack of adherence to the previous treatment was the main reason to start PP1M (*n* = 22, 43.1%) (Table 3). The mean dose of the 1st PP1M injection was 132.6 mg—with 67.4% of patients receiving 150 mg at 1st injection—and a mean steady dose of 120. About 1 mg—only 5.9%—had a steady dose lower than 100 mg. All patients received PP1M at a 4-week interval.

**TABLE 3:** Treatment with palmitate 1-month formulation (PP1M) at the start of the observation period.

	Treatment with PP1M	
	n (%)	nTrts
**Start of observation period with PP1M**		
Reason to start PP1M treatment, n (%)		
Lack of adherence of the previous treatment	22 (43.1%)	−
Lack of efficacy of the previous treatment	9 (17.6%)	−
Lack of efficacy and adherence of the previous treatment	8 (15.7%)	−
Safety or tolerability reasons of the previous treatment2	3 (5.9%)	−
Patient choice	2 (3.9%)	−
Unknown	5 (9.8%)	−
Other	2 (3.9%)	−
**Dose of 1st PP1M injection (mg)**		
N	46	−
Mean (SD)	132.61 (25.75)	−
75 mg	2 (4.4%)	
100 mg	13 (28.3%)	
150 mg	31 (67.4%)	
**Steady dose of PP1M (mg)**		
N	51	−
Mean (SD)	120.10 (28.73)	−
75 mg	3 (5.9%)	
100 mg	27 (52.9%)	
150 mg	20 (39.2%)	
200 mg	1 (2.0%)	
**Frequency of administration, n (%)**		
4-week interval	51 (100.0%)	−

nTrts, number of treatments; PP1M, paliperidone palmitate 1-month formulation.

Only 21.6% of the patients with PP1M at the start of the observation period had a dose change, to a median dose of 150 mg administered in a 4-week interval for most of the sample (93.3%), of whom around 60% had at least 150 mg. Lack of efficacy was the reason to change in 46.7% of the patients, while only two patients (3.9%) discontinued PP1M (lack of adherence and patient’s choice; Table 4).

**TABLE 4:** Palmitate 1-month formulation treatment dose changes and discontinuation at the start of the observation period.

	Treatment with PP1M	
	n (%)	nTrts
**PP1M – dose changes**		
Prescribed dose changed, n (%)		
Yes	11 (21.6%)	−
Dose (mg)		
N	−	15
Median	−	150.00
Min-Max	−	50.00−200
50 mg	2* (13.3%)	
75 mg	3* (20.0%)	
100 mg	1* (6.7%)	
150 mg	6* (40.0%)	
200 mg	3* (20.0%)	
**Frequency of administration, n (%)**		
3-week interval	−	1 (6.7%)
4-week interval	−	14 (93.3%)
**Reason for dose adjustment, n (%)**		
Lack of efficacy	−	7 (46.7%)
Safety or tolerability reasons	−	1 (6.7%)
Patient’s choice	−	1 (6.7%)
Unknown	−	4 (26.7%)
Other	−	2 (13.3%)
**PP1M – discontinuation**		
**Patient discontinued treatment with PP1M during these 12 months, n (%)**		
Yes	2 (3.9%)	−
**Reason for discontinuation of PP1M treatment, n (%)**		
Lack of adherence	1 (50.0%)	−
Patient’s choice	1 (50.0%)	−

nTrts, number of treatments; PP1M, paliperidone palmitate 1-month formulation; *these proportions correspond to the number of dose changes.

Almost 55% of the patients were concomitantly treated with OAP during PP1M treatment, with a cumulative treatment mean duration of 176 days. Of the 72% of patients with concomitant psychotropic medication during treatment with PP1M, 97.2% received anxiolytics/hypnotics, and 40.0% received antidepressants (Supplementary Table 2).

### Clinical outcomes and healthcare resource utilization

**FIGURE 2:**
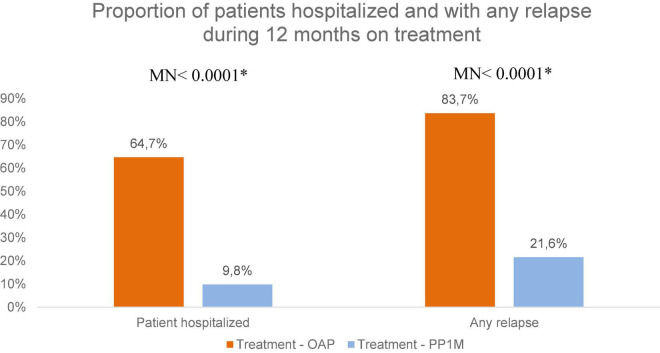
Comparison between treatment conditions for the proportion of patients hospitalized and the occurrence of any relapse during 12 months of treatment. *McNemar test.

There were 33 patients (64.7%) hospitalized during OAP treatment—from a total of 37 hospitalizations—and 5 patients (9.8%) hospitalized during PP1M treatment—from a total of seven hospitalizations (Figure 2 and Supplementary Table 3). The difference in the proportion of hospitalizations between both periods was statistically significant (*p* < 0.0001). The reduction in the proportion of hospitalizations between the two periods was −54.9%, 95%CI = [−70.6%; −39.2%] (Figure 2 and Supplementary Table 3).

**FIGURE 3:**
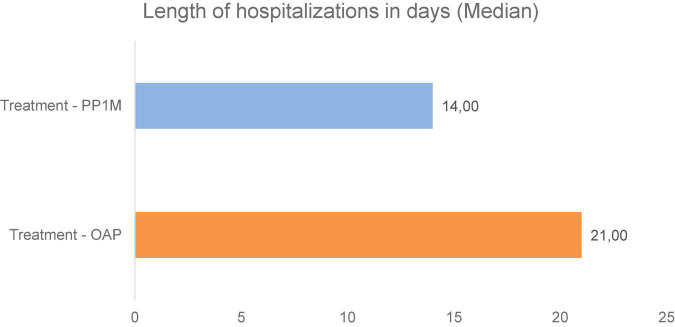
Comparison between treatment conditions for a median length of hospitalizations (in days) during 12 months of treatment.

The mean number of hospitalizations reported during OAP treatment was 0.7 and 0.1 during PP1M treatment. The decrease in hospitalizations between OAP and PP1M periods was statistically significant (−0.59, 95%IC = [−0.80; −0.38]; *p* < 0.0001). To ensure that the duration of OAP treatment was accounted for (Figure 3), an adjusted model for mean change estimates during PP1M treatment showed similar trends, with a statistically significant reduction (−0.44, 95%CI = [−0.84; −0.04]). The leading cause of hospitalizations during both study periods was worsening symptomatology (Supplementary Table 3).

The median length of hospitalizations was 21.0 days for OAP and 14.0 days for PP1M. When considering only the total number of hospitalized patients with complete data (32 in OAP and 5 in the PP1M treatment period), the median cumulative length of hospitalizations was of 21.0 and 22.0 days, respectively.

The sensitivity analysis for the hospitalization rate (including the hospitalization lengths as time at risk) found a hospitalization rate of 0.725 patient-years for OAP and 0.138 patient-years for PP1M treatment (Supplementary Table 3).

In the OAP period, a hospitalization rate of 0.760 patient-years was obtained, which means that per year, 760 out of 1,000 patients were hospitalized. In the PP1M period, 139 out of 1,000 patients were hospitalized per year (hospitalization rate: 0.139 patient-year). The incidence rate ratio was 0.183 indicating a lower rate in the PP1M period (Supplementary Table 3).

The sensitivity analysis was conducted for the primary endpoint and provided no noticeable changes in hospitalization-related findings (Supplementary Table 4). The mirror point were treatment initiation, for patients that started PP1M in the community, and date of discharge, for those that started PP1M during a hospitalization.

Generalized estimating equation models were performed for hospitalization counts and hospitalization prevalence (Supplementary Table 5).

The initial model included sex, age, type of treatment, number of OAPs, duration of OAPs, and use of concomitant psychotropic medication. In both final models, only the type of treatment was statistically significant, leading to a lower rate of hospitalization (IRR = 0.183, 95%CI = [0.082; 0.411]) and a lower risk of hospitalization (OR = 0.059, 95%CI = [0.020; 0.179]) in the PP1M period (Supplementary Table 5).

**FIGURE 4:**
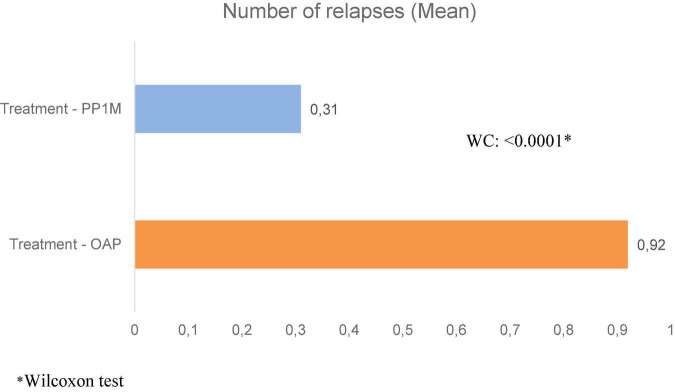
Comparison between samples for the mean number of relapses over the observation period—12 months on treatment. *Wilcoxon test.

Relapses were reported by 83.7% of patients in treatment with OAP and by 21.6% in treatment with PP1M (Figure 2). The difference between these proportions was statistically significant (−61.9%, 95%CI = [−75.3%; −48.5%]; *p* < 0.0001). Also, the difference in the mean number of relapses between both periods was statistically significant (−0.60, 95%CI = [−0.82; −0.38]; *p* < 0.0001) (Figure 4). The main cause of relapse in patients in treatment with OAP was psychiatric hospitalization due to worsening symptomatology (not for social reasons) (80.0% of the relapses), whereas, in patients in treatment with PP1M, the most frequent cause of relapse was an increase in the level of psychiatric care required by the subject (50.0% of the relapses).

Table 5 presents the results for other healthcare resources during the treatment period. A total of 10 (21.7%) patients had at least one emergency psychiatric visit during the treatment with OAP and 4 (8.5%) patients for the duration of treatment with PP1M. The difference in emergency psychiatric visits was not statistically significant (*p* = 0.1797). The mean number of emergency psychiatric visits was, therefore, similar in both periods (approximately 0.2) (*p* = 0.4648).

**TABLE 5:** Other health resources associated with clinical outcomes.

	Treatment with OAP	Treatment with PP1M	*P*-value
**Any emergency psychiatric visit (without hospitalization)−12 months on treatment, n (%)**			
No	36 (78.3%)	43 (91.5%)	MN: 0.1797
Yes	10 (21.7%)	4 (8.5%)	
Total	46	47	
Change in proportions between periods, 95%CI according GEE model	−12.8% [−25.5%; −0.1%]		
**Number of emergency psychiatric visits**			
N	45	47	WC: 0.4648
Mean (SD)	0.24 (0.53)	0.17 (0.64)	
Mean change, 95%CI, according GEE model	−0.07 [−0.27; 0.12]		
**Any outpatient psychiatric consultations, n (%)**			
No	3 (6.0%)	2 (3.9%)	MN > 0.9999
Yes	47 (94.0%)	49 (96.1%)	
Total	50	51	
Change in proportions between periods, 95%CI according GEE model	2.0% [−4.7%; 8.8%]		
**Number of outpatient psychiatric consultations**			
N	50	51	WC: <0.0001
Mean (SD)	4.66 (3.76)	8.33 (6.18)	
Mean change, 95%CI, according GEE model	3.54 [2.15; 4.94]		
**Any relapse−12 months on treatment, n (%)**			
No	8 (16.3%)	40 (78.4%)	MN: <0.0001
Yes	41 (83.7%)	11 (21.6%)	
Total	49	51	
Change in proportions between periods, 95%CI according GEE model	−61.9% [−75.3%; −48.5%]		
**Number of relapses**			
N	49	51	WC: <0.0001
Mean (SD)	0.92 (0.49)	0.31 (0.71)	
Mean change, 95%CI, according GEE model	−0.60 [−0.82; −0.38]		
**Cause of relapse (a), n (%)**			
Psychiatric hospitalization due to worsening symptomatology (not for social reasons) as determined by the investigator	36 (80.0%)	6 (37.5%)	
An increase in the level of psychiatric care required by the subject	8 (17.8%)	8 (50.0%)	
Deliberate self-injury, suicidal or homicidal ideation that is clinically significant as determined by the investigator, or violent behavior resulting in clinically significant injury to another person or property damage.	1 (2.2%)	2 (12.5%)	
Total	45	16	

OAP, oral antipsychotics; PP1M, paliperidone palmitate; 95% CI, 95% confidence interval; MN, McNemar test; WC, Wilcoxon test. (a) Percentages were calculated based on the total number of reported relapses.

Almost all patients reported at least one outpatient psychiatric consultation both in the treatment with OAP and PP1M (respectively, 94.0% and 96.1%, *p* > 0.9999). However, the mean number of outpatient psychiatric consultations was 4.7 with OAP and 8.3 with PP1M and the mean change between treatment periods was statistically significant (3.54, 95%CI = [2.15; 4.94]; *p* < 0.0001).

Safety reporting requirements were followed and Serious Adverse Events (SAEs) were not identified during the observation period of the study.

## Discussion

PSIPROSPER, an observational, multicenter, retrospective, study, set out to assess the potential of including PP1M in the treatment of schizophrenia and its impact on clinical outcomes and healthcare resource utilization, as well as to characterize the patients in routine clinical practice. This study followed a similar mirror study methodology approach as the one used in two previous studies (32, 33). Overall, the results suggest that PP1M promotes better clinical outcomes and less resource utilization compared to the standard of care in which it is not included. Results show a statistically significant reduction of hospitalizations between OAP and PP1M periods in favor of PP1M, as well as a significantly lower risk and rate of hospitalizations. These results coincide with previous research suggesting that PP1M achieves better results in reducing hospitalization, which is even clearer in rehospitalizations in patients treated in monotherapy (22). The same trend was observed for both the presence of relapses and the number of relapses in the observation period, which were significantly lower for the PP1M treatment. Again, these are aligned with the literature and attest to the good clinical outcomes of PP1M (23, 24). The only parameter in which healthcare resources are higher for PP1M was the number of outpatient psychiatric consultations. This may be related to fewer hospitalizations and relapses in the PP1M treatment and may be explained with routine follow-up of patients under treatment. The present results should be carefully analyzed by healthcare decision-makers as the strain on public healthcare services and budget constraints pose new challenges. Also, PP1M helps to address the current unmet needs of patients with schizophrenia in a pandemic context which can be seen as particularly challenging. The correct management of the symptoms could avoid unnecessary and burdensome hospitalizations, as well as provide better overall outcomes for patients, caregivers, and society as a whole. The costs of schizophrenia are significant and better therapeutic is crucial to stemming the rise in costs (12).

The improvements observed here, although measured differently from some previous publications on PP1M, show some positive and relevant clinical outcomes of including this therapeutic. Although a part of the sample was on concomitant therapy with OAP, the data do not provide the duration. Therefore, the results seem aligned with other studies in different countries (26, 32). The same can be said of the tolerability and safety, as the number of discontinuations is very low and corroborates previous research (32, 34), some of them with a very similar mirror design. Also, the outcomes in the hospital resources used to follow the trend of similarly designed studies in other countries, in which a significant decrease in resources was found (35). Concerning measures of treatment adherence and compliance, and although this was measured in the current study considering the proportion of patients that discontinued PP1M—a very low proportion—results follow previously published evidence (36).

The study covered the most relevant regions in Portugal and provides a robust depiction of the clinical setting and treatment of these patients. The 20 sites are regional reference public hospitals with specialized care psychiatric units. The investigators’ clinical decisions were independent of the objectives and scope of the study and were made in the sole interest of the patients. This is relevant since recent changes to the payment scheme in Portugal, in which antipsychotics are provided for free if prescribed by psychiatrists in public hospitals, are expected to impact prescribing trends. In this study, the sample was mostly composed of men between the ages 20 and 55 years, in line with previous data on such population that shows an increased prevalence in men (3, 4). The mean disease duration (4.9 years) at the start of treatment with PP1M and the median time between first symptoms and diagnosis (1.1 years) are also aligned with expectations. However, the median time from first symptoms to diagnosis is still higher than desirable, significantly delaying the start of the treatment, which negatively impacts treatment outcomes (14–16). Misdiagnosis usually happens due to the diagnosis of a myriad of other mental disorders (37). Paranoid-type schizophrenia/paranoid schizophrenia was the most frequent diagnosis, with the most frequent comorbidity being hypercholesterolemia and the most frequently reported psychiatric comorbidities were smoking habits and alcohol/drug abuse. Substance abuse was found to be common in patients with schizophrenia (38, 39). At the inclusion visit, 92.2% of patients were treated with PP1M, with roughly 60% of the patients starting treatment with PP1M as inpatients, which attests to the adaptability of such treatment.

Also of interest are the results on the reasons for the start in both the OAP and PP1M. The most frequent reason to start OAP was the first antipsychotic treatment, while PP1M was initiated in the case of lack of adherence, lack of efficacy of previous treatments, or concomitant lack of adherence and efficacy, which is to be expected due to the therapeutic indication of PP1M. Again, the current results corroborate previous research (20).

Concerning dose adjustment (change), results suggest it to be more frequent for OAP than in PP1M which usually contributes to better treatment compliance and adherence from patients. Dose adjustments mostly occur based on the clinical decision of the psychiatrist and were performed to improve clinical outcomes or to address a lack of adherence. However, the most relevant result concerns the proportion of patients—only two—that discontinued PP1M treatment for the duration of the observation period. This is in stark contrast with the proportion of patients that start another OAP treatment, which had a much higher need for treatment adjustment. Again, stabilizing treatment can provide interesting gains in clinical outcomes (17).

### Limitations

Despite the inherent limitations associated with observational retrospective studies, this design is appropriate for measuring the outcomes of interest.

The consecutive inclusion of the eligible patients, as they attended their routine medical appointments allowed to minimize the sampling bias since all patients fulfilling the study inclusion/exclusion criteria were enrolled until adequate sample size was achieved. This implies, on the other hand, that the patients were not randomized, which was to expect based on the observational design of the study. The decision to start PP1M was exclusively based on the attending psychiatrist’s decision and clinical evaluation based on routine appointments with the patients. This increases the heterogeneity of the sample and the profile of the patients included, which may be identified as a limitation of the current study. Additionally, in the mirror-image design, each patient serves as his/her own control, thus avoiding the need to adjust for selection bias. However, a larger sample size would allow a more complete depiction of the clinical setting in Portugal. As such, missing data limit the potential for inferences in some variables—time from first symptoms to treatment initiation, duration of disease at the start of PP1M, treatment duration at the start of the observation period, and anthropometric characterization—other than the primary objectives.

The sample size was negatively impacted due to recruitment constraints not foreseen in the feasibility. Study sites had higher recruitment expected goals. However, since a significant part of the patients in Portugal did not remain in OAP treatment for 1 year before switching to PP1M, or were under treatment with other LAIs before switching, recruitment was limited.

Also, it must be pointed out that although the number of patients in the combination group (PP1M + OAP) is significantly larger than the monotherapy group, we cannot assume that the patients were under treatment, for the full duration of the PP1M follow-up, with OAP. Thus, the inclusion of PP1M is still the most plausible explanation for the results. However, as suggested in the literature, it is difficult to isolate the effect of a specific treatment, especially in a real-world setting (40).

### Further research

To enhance the potential for generalization of such results, a broader sample should be considered in future studies, although data collection is complicated in the target population. Also of interest is to consider a comparison between patients with treatment-resistant schizophrenia and those without to measure the added economic and humanistic burden—not only in healthcare resource utilization but also in a broader societal perspective. Finally, pre and post-inclusion adherence of PP1M was collected in this study from reason to treatment change. Regardless, using a specific standardized instrument would be of added value in future studies, as this is a relevant parameter in the scope of the treatment of this condition.

## Conclusion

Schizophrenia is a complex, chronic, and burdensome disease in which treatment is essential to provide patients with a certain sense of normality, to improve functionality, safety, and independent living. Current results provide a sound depiction of the reality in Portugal and of the added value of including PP1M in treatment protocols, as measured in both clinical outcomes and resource utilization in patients with schizophrenia. The positive impact of PP1M in the rate and number of hospitalizations, relapses, and overall lower estimated risk of hospitalizations attests to the significant clinical outcomes generated by this treatment option. Although including PP1M in the treatment protocol has a direct impact on costs, the clinical improvement and resource-saving outcomes from such inclusion could be beneficial to patients and the National Health System. The current methodology provided a robust analysis of the outcomes and can be further exploited in future research with broader samples and a longer follow-up period. These results should inform all relevant stakeholders—from physicians to patients/caregivers to healthcare decision-makers—on the significant added value of PP1M from both a clinical and healthcare resource-saving, especially in a context where resources are limited, and public health services are stretched to their limits.

## Data availability statement

The raw data supporting the conclusions of this article will be made available by the authors, without undue reservation.

## Ethics statement

The study protocol was reviewed and approved by an Ethics Committee at each study site: Centro Hospitalar Tâmega e Sousa, Centro Hospitalar Lisboa Ocidental, Hospital de Braga, Hospital Garcia de Orta, Hospital de Magalhães Lemos, Centro Hospitalar de Leiria, Centro Hospitalar e Universitário de Coimbra, E.P.E., Centro Hospitalar Tondela Viseu, Centro Hospitalar Universitário do Algarve, Hospital de Santarém, Centro Hospitalar de Vila Nova de Gaia/Espinho, Hospital de Vila Franca de Xira, Unidade Local de Saúde da Guarda, Centro Hospitalar e Universitário de São João, Centro Hospitalar Médio Tejo, Serviço de Saúde da Região Autónoma da Madeira, Hospital do Espírito de Santo de Évora, Centro Hospitalar de Setúbal, Hospital Beatriz Ângelo, and Hospital Prof. Doutor Fernando da Fonseca. All patients provided written informed consent to participate in the study.

## Author contributions

All authors contributed to the conception, design, analysis, and interpretation of data and involved in writing the manuscript and reviewing drafts, as well as approval of the final manuscript version.
