# Supplementary material for: Results from PSIPROSPER: A multicenter retrospective study to analyze the impact of treatment with paliperidone palmitate 1-month on clinical outcomes and hospital resource utilization in adult patients with schizophrenia in Portugal
Source: Front Psychiatry. 2022 Nov 1;13:992256. doi: 10.3389/fpsyt.2022.992256 (PMC9663469; doi:10.3389/fpsyt.2022.992256)
Supplement: Supplementary file 1 [file Data_Sheet_1.docx]

Supplementary Table 1 – Comorbidities and psychiatric comorbidities at the start of the observation period

|  | Total (n=51) |
| --- | --- |
| Comorbidities, n (%) |  |
| Hypercholesterolemia | 14 (35.0%) |
| Hyperprolactinemia | 6 (21.4%) |
| Obesity | 6 (17.6%) |
| Hypertension | 4 (11.1%) |
| Metabolic syndrome | 3 (8.8%) |
| Diabetes | 3 (7.5%) |
| Galactorrhea | 1 (3.4%) |
| Psychiatric comorbidities, n (%) |  |
| Smoking habits | 20 (48.8%) |
| Past event | 3 (15.0%) |
| Current event | 17 (85.0%) |
| Alcohol and drug abuse | 20 (43.5%) |
| Past event | 14 (70.0%) |
| Current event | 6 (30.0%) |
| Depression | 9 (20.5%) |
| Past event | 7 (77.8%) |
| Current event | 2 (22.2%) |
| Suicide ideation, attempted suicide or non-fatal suicide behaviour | 8 (18.6%) |
| Past event | 8 (100%) |
| Current event | 0 |
| Sexual dysfunction | 5 (19.2%) |
| Past event | 3 (60.0%) |
| Current event | 2 (40.0%) |
| Neurotic, stress-related and somatoform disorders | 5 (11.9%) |
| Past event | 3 (60.0%) |
| Current event | 2 (40.0%) |
| Other mental disorders | 5 (11.4%) |

Supplementary Table 2 – Concomitant OAP treatment and psychotropic medication in patients with PP1M treatment at the start of the observation period

|  | Treatment with PP1M | | | |
| --- | --- | --- | --- | --- |
|  | n (%) | | nTrts | |
| PP1M – Concomitant OAP treatments |  | |  | |
| Patient received also treatment with OAP during PP1M treatment, n (%) | | |  | |
| No | 22 (43.1%) | | - | |
| Yes | 28 (54.9%) | | - | |
| Unknown | 1 (2.0%) | | - | |
| Ongoing |  | |  | |
| No | - | | 42 (67.7%) | |
| Yes | - | | 20 (32.3%) | |
| Cumulative duration of OAP treatments during PP1M treatment (days) | | |  | |
| N | 35 | | - | |
| Mean (SD) | 176.77 (172.33) | | - | |
| Concomitant psychotropic medication | |  | |  |
| Any concomitant psychotropic medication, n (%) | |  | |  |
| No | | 14 (28.0%) | |  |
| Yes | | 36 (72.0%) | |  |
| Total | | 50 | |  |
| If yes, | |  | |  |
| Concomitant psychotropic medication, n (%) | |  | |  |
| Anti-depressants | | 14 (40.0%) | |  |
| Anti-obsessive agents | | 0 | |  |
| Mood stabilizers | | 6 (16.7%) | |  |
| Anxiolytics and hypnotics | | 35 (97.2%) | |  |
| Stimulants | | 0 | |  |
| Anti-panic agents | | 1 (2.8%) | |  |

nTrts: number of treatments; PP1M: paliperidone palmitate 1-month formulation; * these proportions correspond to the number of dose changes.

Supplementary Table 3 – Health resources-hospitalizations (main analysis), hospitalization rate and incidence rate of hospitalization for the comparison between treatment with OAP and PP1M

|  | Main analysis | | |
| --- | --- | --- | --- |
|  | Treatment - OAP | Treatment - PP1M | p-value |
| Patient hospitalized during 12 months on treatment, n (%) |  |  |  |
| Yes | 33 (64.7%) | 5 (9.8%) | MN< 0.0001 |
| Change in proportions between periods, 95%CI according GEE model | -54.9% [-70.6%; -39.2%] |  |  |
| Schizophrenia-related hospitalizations |  |  |  |
| N | 51 | 51 | WC: <0.0001 |
| Mean [95%CI] (SD) | 0.73 [0.56; 0.89](0.60) | 0.14 [0.00; 0.28](0.49) |  |
| Mean change, 95%CI, GEE model | -0.59 [-0.80; -0.38] |  |  |
| Mean change - GEE model (adjusted by duration of OAP treatments during PP1M) | -0.44 [-0.84; -0.04] |  |  |
| Reason for hospitalization a), n (%) |  |  |  |
| Worsening psychiatric symptomatology | 36 (97.3%) | 6 (85.7%) |  |
| Other | 1 (2.7%) | 1 (14.3%) |  |
| Total | 37 | 7 |  |
| Length of hospitalizations (days) b) |  |  |  |
| N | 36 | 7 |  |
| Median (Min-Max) | 21.00 (10.00-57.00) | 14.00 (3.00-45.00) |  |
| Cumulative length of hospitalizations (days) | |  |  |
| N | 32 | 5 | NAc) |
| Median | 21.00 (10.00-75.00) | 22.00 (3.00-50.00) |  |
| Mean change, 95%CI, GEE model | 0.29 [NAc)] |  |  |
| Hospitalization rate per patient-years | 0.760 | 0.139 |  |
| Hospitalization rate per patient-years – sensitivity analysis | 0.725 | 0.138 |  |
| Hospitalization rate and incidence rate ratio of hospitalization | | | |
| Number of hospitalizations | 37 | 7 |  |
| Incidence rate | 0.760 | 0.139 |  |
| Incidence rate ratio of hospitalization d) | 0.183 [0.082; 0.411] |  |  |
| Note: In the main analysis the mirror point of the study was set at 8 days after treatment initiation for patients that started PP1M in the community (outpatients) and for those that started PP1M during a hospitalization (inpatients). Hospitalizations started up to 8 days after PP1M initiation were considered in treatment with OAP period. In the sensitivity analysis the mirror point of the study was: treatment initiation for patients that started PP1M in the community (outpatients) and date of discharge for those that started PP1M during a hospitalization (inpatients). | | | |
| OAP: Oral antipsychotics; PP1M: Paliperidone Palmitate; 95%CI: 95% confidence interval; MN: McNemar test; WC: Wilcoxon test; NA: Not applicable. | | | |
| a) Percentages were calculated based on total number of reported hospitalizations. b) Descriptive statistics were calculated based on total number of reported hospitalizations. c) Due to the small sample size pvalue and 95%CI were not presented. d) Incidence rate ratio was obtained from GEE with Poisson regression without any predictors. e) Percentages were calculated based on total number of reported hospitalizations. c) Due to the small sample size pvalue and 95%CI were not presented. | | | |

Supplementary Table 4 –Health resources-hospitalizations (sensitivity analysis) for the comparison between treatment with OAP and PP1M

|  | Main analysis | | |
| --- | --- | --- | --- |
|  | Treatment - OAP | Treatment - PP1M | p-value |
| Health resources: hospitalizations Sensitivity analysis | | | |
| Number of schizophrenia-related hospitalizations |  |  |  |
| N | 51 | 51 | WC: <0.0001 |
| Mean (SD) | 0.73 (0.60) | 0.14 (0.49) |  |
| Mean change, 95%CI, GEE model | -0.59 [-0.80; -0.38] |  |  |
| Health resources: hospitalizations (sensitivity analysis considering patients treated with PP1M in monotherapy) | | | |
| Number of schizophrenia-related hospitalizations |  |  |  |
| N | 13 | 13 |  |
| Mean (SD) | 0.69 (0.63) | 0.08 (0.28) | WC: 0.0156 |
| Mean change, 95%CI, GEE model | -0.62 [-0.96; -0.28] |  |  |
| Hospitalizations for patients that completed 12 months of PP1M treatment | | | |
| Number of schizophrenia-related hospitalizations |  |  |  |
| N | 49 | 49 |  |
| Mean [95%CI] (SD) | 0.71 [0.55; 0.88] (0.58) | 0.12 [-0.02; 0.26] (0.48) | WC: <0.0001 |
| Mean change, 95%CI, GEE model | -0.59 [-0.80; -0.39] |  |  |
| Length of hospitalizations (days) e) |  |  |  |
| N | 34 | 6 |  |
| Mean (SD) | 23.50 (11.41) | 18.00 (14.72) |  |
| Cumulative length of hospitalizations (days) |  |  |  |
| N | 31 | 4 | NAc) |
| Mean (SD) | 25.77 (14.87) | 27.00 (23.93) |  |
| Mean change, 95%CI, GEE model | 0.29 [NAc)] |  |  |
| Hospitalizations for patients treated with PP1M in monotherapy and completed 12 months of treatment | | | |
| Number of schizophrenia-related hospitalizations |  |  |  |
| N | 13 | 13 | WC: 0.0156 |
| Mean [95%CI] (SD) | 0.69 [0.31; 1.07] (0.63) | 0.08 [-0.09; 0.24] (0.28) |  |
| Mean change, 95%CI, GEE model | -0.62 [-0.96; -0.28] |  |  |
| Note: In the main analysis the mirror point of the study was set at 8 days after treatment initiation for patients that started PP1M in the community (outpatients) and for those that started PP1M during a hospitalization (inpatients). Hospitalizations started up to 8 days after PP1M initiation were considered in treatment with OAP period. In the sensitivity analysis the mirror point of the study was: treatment initiation for patients that started PP1M in the community (outpatients) and date of discharge for those that started PP1M during a hospitalization (inpatients). | | | |
| OAP: Oral antipsychotics; PP1M: Paliperidone Palmitate; 95%CI: 95% confidence interval; MN: McNemar test; WC: Wilcoxon test; NA: Not applicable. | | | |
| a) Percentages were calculated based on total number of reported hospitalizations. b) Descriptive statistics were calculated based on total number of reported hospitalizations. c) Due to the small sample size pvalue and 95%CI were not presented. d) Incidence rate ratio was obtained from GEE with Poisson regression without any predictors. e) Percentages were calculated based on total number of reported hospitalizations. c) Due to the small sample size pvalue and 95%CI were not presented. | | | |

Supplementary Table 5 – Generalized estimating equation (with Poisson distribution) for hospitalization counts and hospitalization

|  |  |  |  |  |  |
| --- | --- | --- | --- | --- | --- |
| Hospitalization counts | | | | | |
|  | | Initial model | | Final model | |
|  | | IRR [95%CI] | p-value | IRR [95%CI] | p-value |
| Sex | |  |  |  |  |
| Male | | 0.995 [0.406; 2.440] | 0.9911 | - | - |
| Female | | Ref. |  | - | - |
| Age | | 0.989 [0.955; 1.024] | 0.5183 | - | - |
| Type of treatment | |  |  |  |  |
| OAP | | Ref. |  | Ref. | - |
| PP1M | | 0.300 [0.103; 0.872] | 0.0271 | 0.183 [0.082; 0.411] | <0.0001 |
| Risk factors: | |  |  |  |  |
| Number of OAPs | | 1.092 [0.899; 1.326] | 0.3762 | - | - |
| Duration of OAPs | | 1.003 [0.997; 1.008] | 0.3899 | - | - |
| Concomitant psychotropic medication | |  |  |  |  |
| Yes | | 2.023 [0.826; 4.954] | 0.1229 | - | - |
| No | | Ref. |  | - | - |
| Hospitalization | | | | | |
|  | | Initial model | Final model |  |  |
|  | | IRR [95%CI] | p-value | IRR [95%CI] | p-value |
| Sex | |  |  |  |  |
| Male | | 0.747 [0.173; 3.221] | 0.696 | - | - |
| Female | | Ref. |  | - | - |
| Age | | 0.981 [0.923; 1.042] | 0.531 | - | - |
| Type of treatment | |  |  |  |  |
| OAP | | Ref. |  | Ref. | - |
| PP1M | | 0.300 [0.103; 0.872] | 0.0271 | 0.183 [0.082; 0.411] | <0.0001 |
| Risk factors: | |  |  |  |  |
| Number of OAPs | | 1.092 [0.899; 1.326] | 0.3762 | - | - |
| Duration of OAPs | | 1.003 [0.997; 1.008] | 0.3899 | - | - |
| Concomitant psychotropic medication | |  |  |  |  |
| Yes | | 2.023 [0.826; 4.954] | 0.1229 | - | - |
| No | | Ref. |  | - | - |
| OAP: Oral antipsychotics; PP1M: Paliperidone Palmitate; IRR: Incidence Rate Ratio. | | | | | |
| Notes: Dose changes were not considered for the number of OAPs. A total of 80 observations were used in the initial model and 102 in the final model. | | | | | |

Supplementary table 6. Site and primary investigator list

| Site # | Site Name | Name | Study Role |
| --- | --- | --- | --- |
| 01 | 01 - Centro Hospitalar Tâmega e Sousa | Orlando von Doellinger | Primary Investigator |
| 02 | 02 - Centro Hospitalar Lisboa Ocidental | Joaquim Gago | Primary Investigator |
| 03 | 03 - Hospital de Braga | Pedro Morgado | Primary Investigator |
| 04 | 04 - Hospital Garcia de Orta | Gonçalo Sobreira | Primary Investigator |
| 05 | 05 - Hospital de Magalhães Lemos | Liliana Correia Castro | Investigator |
| 06 | 06 - Centro Hospitalar de Leiria | Cláudio Laureano | Primary Investigator |
| 07 | 07 - Centro Hospitalar e Universitário de Coimbra, E.P.E. | Nuno Madeira | Primary Investigator |
| 08 | 08 - Centro Hospitalar Tondela Viseu | Marta Silva Gouveia | Investigator |
| 09 | 09 - Centro Hospitalar Universitário do Algarve | Marco Oliveira | Primary Investigator |
| 10 | 10 - Hospital de Santarém | Elisabete Sêco | Primary Investigator |
| 11 | 11 - Centro Hospitalar de Vila Nova de Gaia/ Espinho | Georgina Lapa | Primary Investigator |
| 12 | 12- Hospital de Vila Franca de Xira | Amílcar Silva-dos-Santos | Primary Investigator |
| 13 | 13 - Unidade Local de Saúde da Guarda | António Pissarra da Costa | Primary Investigator |
| 14 | 14 - Centro Hospitalar e Universitário de São João | Susana Fonseca | Primary Investigator |
| 15 | 15 - Centro Hospitalar Médio Tejo | Luisa Paiva Delgado | Primary Investigator |
| 16 | 16 - Serviço de Saúde da Região Autónoma da Madeira | Joana Gomes | Investigator |
| 17 | 17 – Hospital do Espírito de Santo de Évora | João Pedro | Primary Investigator |
| 18 | 18 - Centro Hospitalar de Setúbal | António Gamito | Primary Investigator |
| 19 | 19 - Hospital Beatriz Ângelo | Claudia Mota Pinto | Primary Investigator |
| 20 | 20 - Hospital Prof. Doutor Fernando da Fonseca | Raquel Ribeiro | Primary Investigator |
